# Endodontic Sealers Modified with Silver Vanadate: Antibacterial, Compositional, and Setting Time Evaluation

**DOI:** 10.1155/2019/4676354

**Published:** 2019-05-09

**Authors:** Ana Beatriz Vilela Teixeira, Caroline de Carvalho Honorato Silva, Oswaldo Luiz Alves, Andréa Cândido dos Reis

**Affiliations:** ^1^Dental Materials and Prosthesis Department, Ribeirão Preto School of Dentistry, University of São Paulo, 14040-904 Ribeirão Preto, SP, Brazil; ^2^Laboratory of Solid State Chemistry, Institute of Chemistry, University of Campinas (Unicamp), 13083-970 Campinas, SP, Brazil

## Abstract

The incorporation of nanoparticles into endodontic sealers aims at increasing antimicrobial activity of the original material.* Aim. *The aim of this study is to incorporate the nanostructured silver vanadate decorated with silver nanoparticles (AgVO_3_, at 2.5%, 5%, and 10%) into three endodontic sealers and evaluate the antibacterial activity of freshly sealers, surface topography and chemical composition, and setting time.* Material and Methods*. The AgVO_3_ was incorporated into AH Plus, Sealer 26, and Endomethasone N at concentrations 0%, 2.5%, 5%, and 10% (in mass). The antibacterial activity of freshly sealers was assessed by direct contact with* Enterococcus faecalis *and CFU/mL count (n=10), surface topography, and chemical composition were measured by SEM/EDS, and the setting time was measured by Gillmore needle (n=10). The Kruskal-Wallis and Dunn statistical tests were applied (*α*=0.05).* Results.* All groups of sealers evaluated inhibited* E. faecalis* (p>0.05). The incorporation of AgVO_3_ altered the atomic proportions between components of the endodontic sealers, and the percentage of silver (Ag) and vanadium (V) increased proportionally to the concentrations of AgVO_3_. Topography analysis showed differences in components distribution on the surface of the specimens. The sealers incorporated with AgVO_3_ of AH Plus presented a lower setting time than the control group (p<0.05). For Sealer 26 and Endomethasone N, the incorporation of AgVO_3_ increased the setting time in relation to control group (p<0.05).* Conclusions.* The modification of endodontic sealers by AgVO_3_ increased the atomic percentage of Ag and V proportionally to the concentration of the nanomaterial and changed the atomic percentage of the sealer components and setting times. It cannot be affirmed that the AgVO_3_ promote differences in the antimicrobial activity of freshly sealers, and further investigations of the antimicrobial activity of the set sealers should be carried out.

## 1. Introduction

The elimination of bacteria from the root canal is a challenge in endodontic treatment. Even after the combined use of mechanical instrumentation and chemical irrigation, retreatment cases indicate failures in bacterial removal [1]. Species such as the* Enterococcus faecalis* are often found in these cases, due to their resistance and virulence factors [2]. One of the ideal requirements for root filling materials, such as endodontic sealers, is the antimicrobial capacity to assist in the elimination of remaining viable microorganisms in areas of difficult access in the root canal system [3]. The composition of some sealers is inherently antimicrobial, but its effect is short-lived [3]. It is known that the incorporation of antimicrobial agents into endodontic sealers increases this ability [3]. However, studies with sealers modified with these agents have shown varied effects, and provide little information about other properties [1].

The incorporation of micro- and nanoparticles into endodontic sealers promotes a prolonged release of antimicrobial components [1]. Nanometric materials have greater surface area/mass ratios and chemical reactivity, which increases the antimicrobial activity [4, 5]. Nanostructured silver vanadate decorated with silver nanoparticles (AgVO_3_) is a novel antimicrobial nanomaterial for this application. It is composed of vanadium and silver, which synergistically interact with microorganisms through the cell membrane and of the thiol groups present in the enzymes of bacterial metabolism [5]. In addition to presenting the antimicrobial effectiveness of silver nanoparticles, the silver vanadate nanowires solve the dispersion limitation of the silver nanoparticles [5].

The AgVO_3_ incorporated into dental acrylic resin in low concentrations (0.5%; 1%; 2.5%; 5%; and 10%) inhibited the growth of* Candida albicans*,* Streptococcus mutans*,* Staphylococcus aureus*, and* Pseudomonas aeruginosa *[6, 7]. A preliminary study incorporated the AgVO_3_ into endodontic sealers (at concentrations 0%, 2.5%, 5%, and 10%) and showed promising antimicrobial activity against* E. faecalis*,* P. aeruginosa,* and* Escherichia coli* [8], with other tests being necessary to confirm this effect.

This modification with antimicrobial agents into sealers might influence the material's physical properties [1, 9] such as the setting time, which varies with different components, particle sizes, temperature, and humidity [10]. Thus, the determination of the chemical composition and topography of the material allows correlating the physical, antimicrobial, and biological properties.

The chemical composition of these materials is essential for antimicrobial efficacy; however, it may lead to inflammatory reactions to the periapical cells [11, 12]. The silver nanoparticles are reported as low toxicity to human cells; however, this toxicity depends on the concentration incorporated [13, 14]. In the case of AgVO_3_, it was reported that lower concentrations release less silver (Ag) and vanadium (V) ions and avoid risk to patients, since the chemical elements released can be distributed in the body through blood vessels, resulting in cytotoxic effects [15]. The toxicity of AgVO_3_ to human cells is still unknown; however, this nanomaterial was reported as cytotoxic to* Daphnia similis*, an aquatic organism, as a function of Ag [16].

Given this perspective, this study provides an investigation of the antimicrobial activity of three commercial endodontic sealers freshly mixing and incorporated with AgVO_3_, in low concentrations (2.5%, 5%, and 10%), against* Enterococcus faecalis*, and investigated the influence of this incorporation at topography, chemical composition, and setting time of this sealers. The hypothesis was that AgVO_3_ would increase antibacterial activity and would affect topographic, compositional, and setting time of the evaluated sealers.

## 2. Materials and Methods

### 2.1. Sample Preparation

The nanostructured silver vanadate decorated with silver nanoparticles (AgVO_3_) was synthesized according to the method described by Castro et al. [6] and was incorporated into the endodontic sealers AH Plus, epoxy-amine resin based (DENTSPLY DeTrey GmbH, Konstanz, Germany), Sealer 26, calcium hydroxide-based (DENTSPLY, Petrópolis-RJ, Brazil), and Endomethasone N, zinc oxide, and eugenol based (SEPTODONT, Saint-Maur-des-Fossés, France) at concentrations 0% (control group), 2.5%, 5%, and 10%. The powder/base paste of the control group was considered 100%, and for the addition of AgVO_3_, the concentrations of the nanomaterial (2.5%, 5%, and 10%) were subtracted from the total mass. The powder/base paste of the sealers and the concentration of AgVO_3_ amounts were weighed on a precision scale (Micronal S/A, model AB 204, São Paulo, SP, Brazil) and then incorporated into the liquid or catalyst paste. The catalyst paste of AH Plus was weighed at the same proportion of the base paste of the control group, and this proportion was used for modified groups. The same amount of drops used for the control group of the sealers Sealer 26 and Endomethasone N was used for the modified groups (Table 1). The mixing was done on an unpolished glass plate, because the roughness of the plate helps in the dispersion of the granules of the nanomaterial, promoting better incorporation.

### 2.2. Evaluation of Antibacterial Activity

The direct contact test was used to evaluate the inhibitory effect of* Enterococcus faecalis* (ATCC 29212), obtained from a recent culture and standardized in a spectrophotometer (Multiskan GO, Thermo Fisher Scientific, Waltham, MA, USA), with an absorbance of 0.150 at 625 nm wavelength (10^8^ CFU/mL bacteria). The methodology used was based on Zhang et al. with adaptations [17]. The endodontic sealers were prepared in an aseptic environment and 20 *μ*L of each sealer (n = 10) was placed in wells of a 96-well plate with a sterile syringe. Then, 50 *μ*L of the* E. faecalis* suspension was placed on the specimens and the plates were incubated at 37°C for 1 h in a microbiological oven. Fifty microliters of the bacterial suspension were used as positive control and sealers without bacterium were used as negative control. After incubation, 100 *μ*L of sterile Tryptic Soy Broth (TSB) (Difco, Sparks, MD, USA) was added to the wells and gently mixed with a pipette for 1 min. The samples were serially diluted in phosphate buffered saline (PBS), seeded in Trypticase Soy Agar (Difco, Sparks, MD, USA), and incubated at 37°C for 24 h in a microaerophilic environment in anaerobic jars. The colony forming units (CFU) were counted and CFU/mL was calculated. One experiment was performed with 10 samples per group. The CFU/mL values were Log_10_ transformed.

### 2.3. Topographic and Compositional Evaluation

To obtain test specimens, the control and modified endodontic sealers were prepared and inserted in silicone matrices (Ø7.75 x 1.5 mm) (Zetalabor©, Zhermack SpA, Badia Polenise-RO, Italy) and incubated (DeLeo, B2DG) at 37°C for 7 days for complete setting. After, the specimens were fixed on an aluminum stub (Ø10 x 2 mm) and elemental microanalysis was carried out using EDS (IXRF Systems mod. 500 Digital Processing, Houston, USA) coupled to a SEM (ZEISS model EVO 50, Cambridge, United Kingdom) with a 20 kV electron beam, using SE and BSD detectors for topographic and compositional evaluations, respectively. Microanalysis was performed at a working distance of 8.5 mm, Iprobe at 20 nA, and dead time at approximately 30%, using the BSD detector, with a magnification of 300x. To increase the conductivity, specimens were gold-sputtered for 120 s on the BAL-TEC equipment (model SCD 050 Sputter Coater, Fürstentum, Liechtenstein). After obtaining the spectrum, the chemical elements were quantified in atomic percentages (wt%). Micrographs was obtained from the same area of the elemental microanalysis, with the SE detector, at 300, 1k, 5k, 10k, 30k, 50k, and 100k x magnifications.

### 2.4. Setting Time

For the preparation of specimens, the standard ISO 6876 and ADA no. 57 were used. The control and modified endodontic sealers were proportioned, manipulated, and placed in metal rings measuring 10 mm in internal diameter and 2-mm thick (n = 10). The test was performed according to standard ASTM C266 [18] for determining the setting times and under controlled temperature and humidity conditions (37°C ± 1°C and 95%). Two Gillmore needle was used on the surface of the specimens, exerting vertical pressure. For initial setting time a needle of 100 g and active tip of 2.0 mm in diameter were used, and for a final setting time a needle of 456 g and active tip of 1.0 mm were used. The measurements were performed until not leaving a mark on the material's surface and obtained in minutes.

### 2.5. Statistical Analysis

For the analysis of the data, the Kruskal-Wallis test and Dunn's post hoc (*α* = 0.05) were applied using the software SPSS v 20.0 (SPSS, USA).

## 3. Results

### 3.1. Antimicrobial Activity

All test and control endodontic sealers evaluated in this study completely inhibited the growth of* E. faecalis*; no statistical difference was observed between groups (p>0.05) (Table 2).

### 3.2. Topographic and Compositional Evaluation

**Figure 1:**
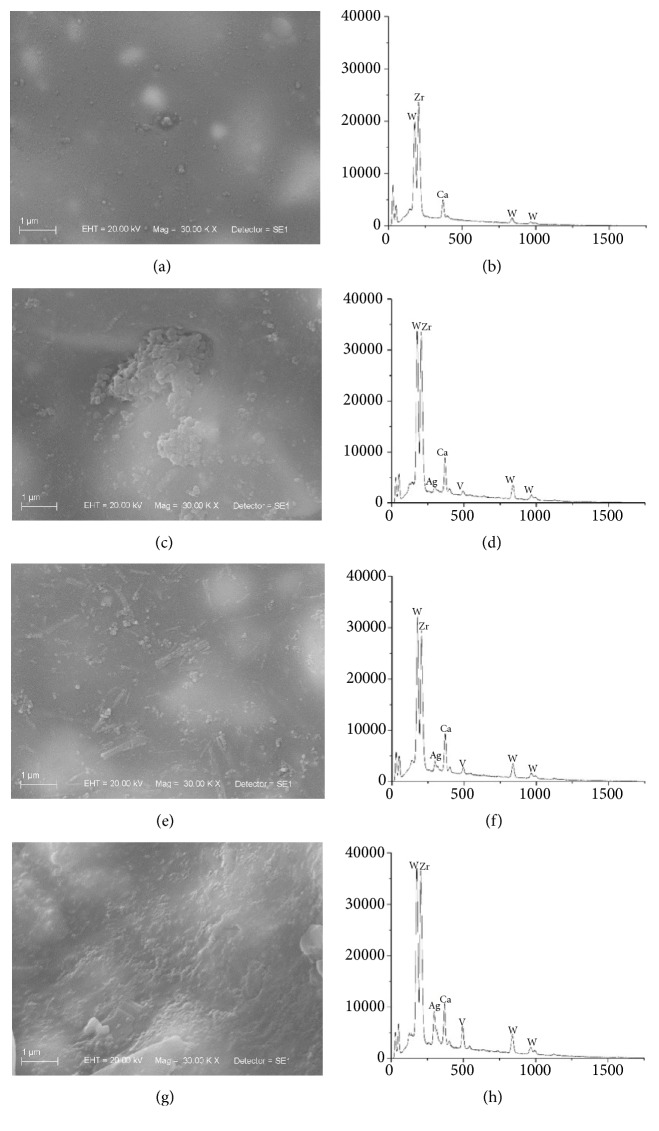
Scanning Electron Microscopy (SEM) micrographs and energy-dispersive X-ray spectroscopy (EDS) analysis of the AH Plus. (a) Control group micrograph. (b) Control group EDS spectrum. (c) 2.5% of AgVO_3_ micrograph. (d) 2.5% of AgVO_3_ EDS spectrum. (e) 5% of AgVO_3_ micrograph. (f) 5% of AgVO_3_ EDS spectrum. (g) 10% of AgVO_3_ micrograph. (h) 10% of AgVO_3_ EDS spectrum.

**Figure 2:**
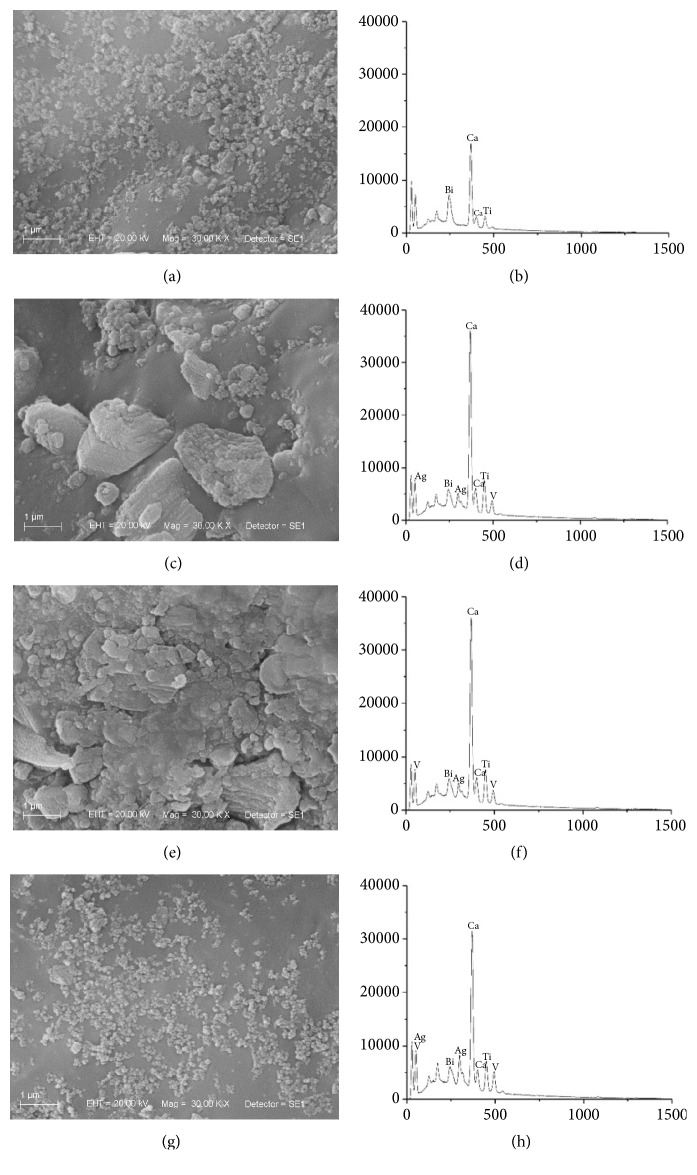
Scanning Electron Microscopy (SEM) micrographs and energy-dispersive X-ray spectroscopy (EDS) analysis of the Sealer 26. (a) Control group micrograph. (b) Control group EDS spectrum. (c) 2.5% of AgVO_3_ micrograph. (d) 2.5% of AgVO_3_ EDS spectrum. (e) 5% of AgVO_3_ micrograph. (f) 5% of AgVO_3_ EDS spectrum. (g) 10% of AgVO_3_ micrograph. (h) 10% of AgVO_3_ EDS spectrum.

**Figure 3:**
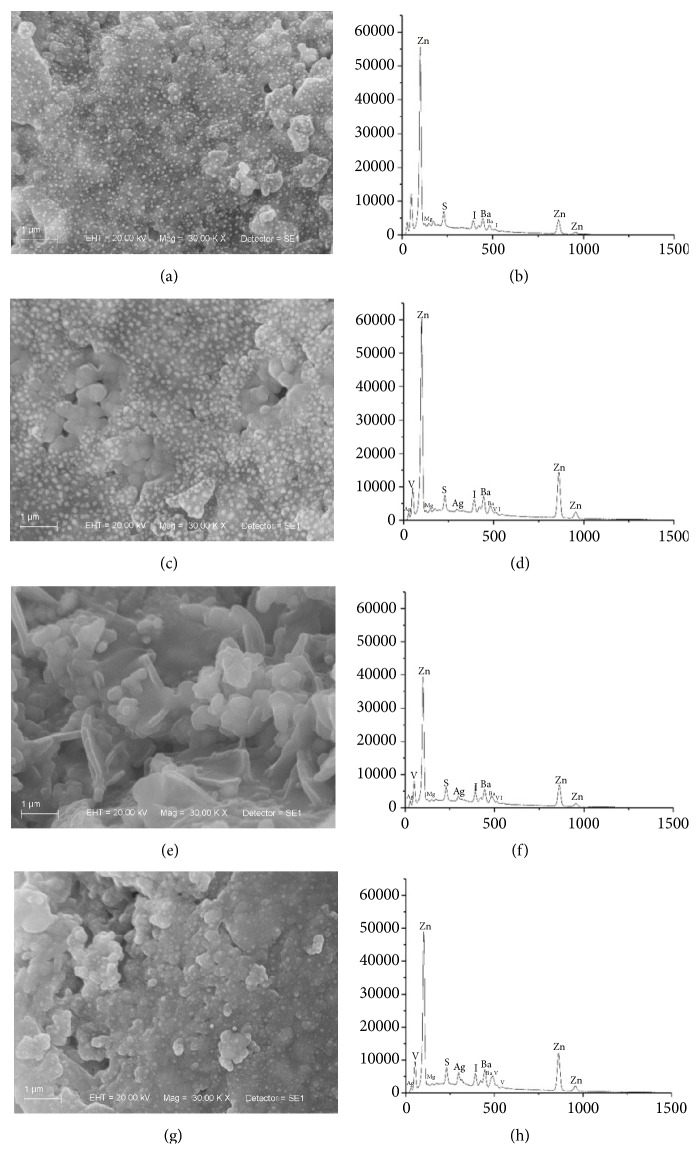
Scanning Electron Microscopy (SEM) micrographs and energy-dispersive X-ray spectroscopy (EDS) analysis of the Endomethasone N. (a) Control group micrograph. (b) Control group EDS spectrum. (c) 2.5% of AgVO_3_ micrograph. (d) 2.5% of AgVO_3_ EDS spectrum. (e) 5% of AgVO_3_ micrograph. (f) 5% of AgVO_3_ EDS spectrum. (g) 10% of AgVO_3_ micrograph. (h) 10% of AgVO_3_ EDS spectrum.

EDS results showed that the addition of AgVO_3_ to the endodontic sealers occurred homogeneously, since the atomic percentage of silver (Ag) and vanadium (V) increased proportionally to the concentrations of AgVO_3_. The incorporation of AgVO_3_ altered the atomic proportions among components of the endodontic sealers, as observed in the AH Plus, that presented a higher atomic percentage of calcium (Ca) and tungsten (W) in the groups modified with AgVO_3_ in relation to the control group. The Endomethasone N presented higher percentage of zinc (Zn) and Sealer 26, a higher percentage of titanium (Ti) (Table 3). In addition, the incorporation of the nanomaterial altered the molecular interactions among sealers components, as observed in the topographic distribution of the sealers in Figures 1, 2, and 3.

### 3.3. Setting Time

The sealers incorporated with AgVO_3_ of AH Plus presented a lower setting time than the control group (p<0.05). For Sealer 26, the incorporation of AgVO_3_ increased the setting time in relation to control group, with a significant difference between of control and the groups with 2.5% and 5% of AgVO_3_ (p<0.05). The group incorporated with 2.5% of the nanomaterial presented the higher set, being statistically different from the 10% group (p<0.05). The Endomethasone N control group presented lower setting time and a significant difference in relation to groups modified with 5% and 10% AgVO_3_ (p<0.05). The group incorporated with 5% of the nanomaterial presented the higher set, being statistically different from the 2.5% group (p<0.05) (Table 4).

## 4. Discussion

The greatest challenge in modifying materials with the purpose of altering their biological, antimicrobial, and/or mechanical properties is obtaining a product that presents stability over time, has a homogeneous incorporation of the additives, and can be easily handled. In this study, AgVO_3_ [5] was incorporated into three freshly mixed endodontic sealers of different compositions to improve antibacterial efficacy, with objective of not affecting the setting time and checking the topographic and composition. The hypothesis of this study was partially accepted, since it was not possible to verify the action of AgVO_3_ on the antimicrobial activity.

All experimental groups freshly mixed inhibited the growth of* E. faecalis*. Al-Shwaimi et al. [19] reports that epoxy resin-based, zinc oxide, and eugenol-based and calcium hydroxide-based sealers presented strong antibacterial effect of freshly mixed against* E. faecalis*; however, this effect decreases after the set of sealers. Thus, the effect against* E. faecalis* observed under the conditions of this study may be due to release of components of sealers during the setting or due to pH changes promoted by them, not being possible to evaluate the influence of AgVO_3_.

The direct contact test used in this study evaluates the effect of the contact between the material and the microorganism tested, allowing the reproducibility and quantification of viable microorganism, overcoming some disadvantages of the agar diffusion test [19]. On the other hand, tests with bacteria planktonic provide preliminary results on the antimicrobial activity of disinfectant agents [20] and depend on the material's ability to dissolve and diffuse their compounds [21]. Thus, it is a necessary future investigation of the antimicrobial activity of the set sealers incorporated with AgVO_3_ in models formed with biofilm and dentin substrate, and the release of antimicrobial components over time should be carried out to investigate the influence of the nanomaterial.

The antimicrobial capacity of endodontic sealers is determined by its composition, which also influences its physical, chemical, and biological behavior [10]. In this study, the topographic and compositional analyses were performed by SEM/EDS. With the SE detector, differences were observed in the surfaces of specimens. Areas rich in light atoms, such as carbon, tend to be darker than areas with elements of higher atomic numbers, such as zinc [22].

The Endomethasone N sealer, independent of the AgVO_3_ concentration, presented pores on its surface, which correspond to the organic phase of its composition. In Figure 3(a), brighter elements on the surface of the control group are observed, which correspond to the inorganic phase. These elements are also present in Endomethasone N + 2.5% AgVO_3_ (Figure 3(c)), and more discreetly just below the surface in Endomethasone N + 10% AgVO_3_ (Figure 3(g)). However, the bright elements were not observed on the surface of Endomethasone N + 5% AgVO_3_ (Figure 3(e)), suggesting that the incorporation of AgVO_3_ may have altered the molecular interactions among components of this sealer. Through compositional analysis (Table 3) we found that the atomic percentage (wt%) of the elements varied with the incorporation of the nanomaterial, and as expected, Ag and V increased with the increasing concentration of AgVO_3_.

Endodontic sealers are in contact with periapical tissues, and surface irregularities favor cell adhesion and influence biocompatibility [10, 23]. Figures 1 and 2 demonstrate different patterns of particle distribution on the surface of the AH Plus and Sealer 26 in the control group compared to AgVO_3_-modified specimens. Through EDS compositional analysis, a uniform and increasing distribution of Ag and V, as well as a variation in wt% of the sealers components with the incorporation of AgVO_3_, was observed.

These results demonstrate the homogeneous incorporation of the nanomaterial (Table 3 and Figures 1, 2, and 3), determining the effectiveness of the mixture proposed in the study. The incorporation of AgVO_3_ may have caused a restructuring of the sealers constituent molecules, resulting in a different material than the original. The SEM/EDS equipment allows evaluating the topography and surface composition of the material, which theoretically must be the same as its inner layers. However, due to the contact with contaminants and the thermodynamic effect of the surface, the superficial composition might be different from the material bulk [23].

Differences between the surface of the material and its inner mass were also observed in the evaluation of the setting time. Although the surface of the specimen is rigid, its inner layer might remain soft for a longer time, demonstrating that setting reactions are complex and depend on the components of the formula, particle size, temperature, and humidity [10]. Gillmore needles are frequently used for determining the setting time; being placed on the surface of the sealers and when their own weight leaves no marks, the set occurred [24].

According to the American National Standards Institute/American Dental Association (ANSI/ADA), the setting time cannot exceed 10% of the time specified by the manufacturer [10, 25]. In this study, the control groups exceeded the recommended setting times, and the incorporation of AgVO_3_ decreased the setting time for the modified groups of AH Plus and delayed for the modified groups of Sealer 26 and Endomethasone N. Studies that evaluated the incorporation of antimicrobials (QPEI, benzalkonium chloride, and iodoformium nanoparticles [26, 27]) into endodontic sealers also showed an increase in setting time. On the other hand, another study also observed a decrease in the setting time of the AH Plus incorporated with amoxicillin [28]. Despite the reduction in time for the set, this time is adequate to the conditions of clinical use.

The setting of a sealer occurs due to the reaction between the two components of the material (powder with liquid or base paste with catalyst paste), as in the reaction between the bisphenol-epoxy resin and hexamethylenetetramine of Sealer 26, for example, [27]. Thus, the addition of particles that do not participate in the reaction of set or polymerization may increase the setting time [9]. This occurred with the incorporation of AgVO_3_ into Sealer 26 and Endomethasone N, interfering at component's proportions and reaction of set, since for the AgVO_3_ addition, the components of the sealers (powder/base paste) were subtracted.

A little delay in hardening may favor the antibacterial activity that is mediated by substances released during the setting of the material [21]; however, this time cannot be very long, because the contact of the material with the periapical tissues may cause irritation and affect the biocompatibility. Moreover, a long time to set may favor the solubility, causing gaps that could be colonized by microorganisms that lead to reinfection [24, 28].

The applied methodology showed a homogeneous incorporation of AgVO_3_ to the evaluated endodontic sealers, demonstrating the viability of the mixture proposed in this study and resulting in a different material from the commercial product. Moreover, the modified sealers were easy to handle and of low cost. Future investigations regarding the preservation of antimicrobial activity overtime, physicochemical properties, and characterization of the chemical composition should be carried out, in association with studies on the biological response of human cells.

## 5. Conclusions

The modification of endodontic sealers by AgVO_3_ increased the atomic percentage of Ag and V proportionally to the concentration of the nanomaterial and changed the atomic percentage of the sealer components and setting times. It cannot be affirmed that the AgVO_3_ promote differences in the antibacterial activity of freshly sealers against* Enterococcus faecalis*, further investigations of the antimicrobial activity of the set sealers incorporated with AgVO_3_ and the release of components over time should be carried out to investigate.

## Figures and Tables

**Table 1:** Quantity of silver vanadate (AgVO_3_) and endodontic sealers used at this study.

Endodontic Sealer	[AgVO_3_]	Powder/Paste A (g)	Powder AgVO_3_ (g)	Liquid (drops)/Paste B (g)
AH Plus	0%	0.1300	-	0.1300
	2.5%	0.1267	0.0033	
	5%	0.1235	0.0065	
	10%	0.1170	0.0130	
Sealer 26	0%	0.0600	-	1 drop
	2.5%	0.0585	0.0015	
	5%	0.0570	0.0030	
	10%	0.0540	0.0060	
Endomethasone N	0%	0.1453	-	2 drops
	2.5%	0.1416	0.0037	
	5%	0.1380	0.0073	
	10%	0.1307	0.0146	

**Table 2:** Colony Forming Unit per milliliter of *Enterococcus faecalis *after direct contact with endodontic sealers incorporated with AgVO_3_ (Log_10_ CFU/mL).

Endodontic sealer	0%	2.5%	5%	10%	Positive control
AH Plus	0^*∗* a^	0 (-0.32; 0.84) ^a^	0 (-0.19; 0.92) ^a^	0^*∗* a^	7.62 (7.56-7.70) ^b^
Sealer 26	0 (-0.20; 0.52) ^a^	0 (-0.18; 0.88) ^a^	0 (-0.16; 0.80) ^a^	0 (0.05; 1.77) ^a^	7.52 (7.43; 7.61) ^b^
Endomethasone N	0 (-0.47; 1.77) ^a^	0^*∗* a^	0^*∗* a^	0 (-0.32; 0.84) ^a^	7.27 (6.95; 7.33) ^b^

Statistical test: Kruskal-Wallis and Dunn's post hoc. Median (confidence interval).  ^*∗*^constant value for all specimens.  ^ab^ Same letters represent statistical similarity in the same line (p>0,05).

**Table 3:** Elements found in endodontic sealers incorporated with AgVO_3_ using energy-dispersive X-ray spectroscopy analysis (EDS) (wt.%).

Endodontic sealer	Elements	0%	2.5%	5%	10%
AH Plus	Zr	68.64	56.54	50.23	44.02
	Ca	18.91	19.82	21.32	16.78
	W	12.36	18.00	18.43	17.41
	Ag	-	3.09	5.53	12.05
	V	-	2.53	4.47	9.73
Sealer 26	Ca	79.55	72.55	71.78	65.20
	Ti	16.28	19.58	18.15	17.21
	Bi	4.16	2.31	2.94	2.52
	Ag	-	2.60	2.04	5.37
	V	-	2.94	5.07	9.69
Endomethasone N	Zn	52.25	73.58	57.67	64.29
	S	20.04	9.61	12.59	9.72
	Ba	11.28	6.87	9.63	8.01
	I	8.39	5.73	7.63	5.75
	Mg	8.01	2.43	6.54	2.54
	Ag	-	1.16	3.77	5.44
	V	-	0.59	2.14	4.21

**Table 4:** Final setting times of endodontic sealers incorporated with different concentrations of AgVO_3_ (min).

Endodontic sealer	0%	2.5%	5%	10%
AH Plus	1926.03 (1926.01; 1926.04) ^a^	1390.06 (1367.80; 1799.26) ^a^	1415.02 (1415.01; 1415.04) ^a^	1415.03 (1415.01; 1415.04) ^a^
Sealer 26	5910.03 (5910.01; 5910.04) ^a^	7069.03 (7069.01; 7069.04) ^ab^	7045.02 (7045.01; 7045.04) ^a^	7009.03 (7009.02; 7009.05) ^b^
Endomethasone N	4470.03 (4356.91; 4583.14) ^a^	20040.02 (20040.01; 20040.04) ^b^	39090.03 (35171.36; 41438.68) ^ab^	34440.00 (30915.33; 34688.72) ^a^

Statistical test: Kruskal-Wallis and Dunn's post hoc. Median (confidence interval).  ^ab^Same letters represent statistical difference in the same line (p<0,05).

## Data Availability

The complete data of colony forming units, atomic percentage of elements of sealers, and setting time used to support the findings of this study are available from the corresponding author upon request.
